# Antibiotics Resistance Genes Screening and Comparative Genomics Analysis of Commensal* Escherichia coli* Isolated from Poultry Farms between China and Sudan

**DOI:** 10.1155/2018/5327450

**Published:** 2018-08-26

**Authors:** Sheikheldin A. Abdelgader, Donglin Shi, Mianmian Chen, Lei Zhang, Hassan M. A. Hejair, Umair Muhammad, Huochun Yao, Wei Zhang

**Affiliations:** ^1^College of Veterinary Medicine, Nanjing Agricultural University, Nanjing 210095, Jiangsu Province, China; ^2^College of Animal Production, Bahri University, Khartoum, Sudan; ^3^College of Food Science and Technology, Nanjing Agricultural University, Nanjing 210095, Jiangsu Province, China

## Abstract

*Escherichia coli *(*E. coli*) strains, from the gut of animals and humans, harbor wide range of drug resistance genes. A comparative study is conducted on the intestinal* E. coli* from fecal samples of healthy chicken from China and Sudan in order to monitor the antimicrobial sensitivity pattern. A number of 250* E. coli *isolates from chicken farms, including 120 from China and 130 from Sudan, were isolated and identified. All isolates were subjected to susceptibility tests against 10 antibiotics and the distribution of antibiotic resistant genes was confirmed by PCR amplification, involving genes such as* ampC, tetA, pKD13, acrA, ermA, ermB, ermC, tetB, mphA, aadA14, aadA1, aac3-1, and aac3- III.* Many isolates were found to exhibit resistance against more than one antibiotic. However, the Chinese isolates showed more antibiotics resistance and resistance genes compared to the Sudanese isolates. For better understanding of the multidrug resistance factors, we conducted whole genome analyses of* E. coli* D107 isolated from China, which revealed that the genome possesses multiple resistance genes including tetracycline, erythromycin, and kanamycin. Furthermore,* E. coli* D4 isolate from Sudan was more sensitive to antibiotics such as erythromycin, tetracycline, and gentamicin. After analysis by RAST and MAUVE, the two strains showed 89% average nucleotide identity. However, the genomes mostly differed at the number of antibiotics-related genes, as the genome of D107 revealed a considerable number of antibiotics resistance genes such as* ermA *and* mphD* which were found to be absent in D4 genome. These outcomes provided confirmation that the poultry farms environment in different countries (China and Sudan) may serve as a potential reservoir of antimicrobial resistance genes and also indicated the evolutionary differences of strains in terms of resistant genes expression.

## 1. Introduction

Antibiotic resistance is one of the upcoming crucial concerns to global health care [1], and the spread of antibiotic-resistant bacteria stands as one of the most dangerous global health care issues to human health [2]. This resistance among bacteria is now recognized to have a considerable effect in rising morbidity, mortality, and costs associated with major public health problems [3]. Increasing antimicrobial resistance problems do not only affect developed countries but also affect nonindustrialized countries, where antibiotics resistance issues are more challenging, because of the lack of well-organized antimicrobial usage policies and the need for optimal hygiene situation and contagion control practices [4]. The use of antimicrobials in animal farm does not only induce the resistance in the pathogens but also produce resistance in the commensal bacterial of individuals or groups [5, 6]. Description of antimicrobial resistance determinants in bacteria at the genetic level plays a critical role in understanding and possibly controlling the resistance [7]. Moreover, it is clear that genetic exchange system and the ability of* E. coli* to transfer and propagate genes between humans and animals may make it a significant vector for the spread of rapidly dispersed resistance genes [8, 9].

Genomes resistant to antimicrobials might have emerged from continuous accumulation of multiple mutations [12]. Hence, whole-genome sequence (WGS) of microorganisms has become an important tool for antibiotics resistance screening and, thus, provides rapid identification of antibiotic resistance mechanisms. Moreover, WGS also enables evaluating the number of mutations and functions of the mutated genes [13, 14]. Sequencing the entire genome is found to be helpful in many antimicrobial applications such as new antibiotics development, diagnostic tests, the management of presently available antibiotics, and clarifying the factors promoting the emergence and resistance of pathogenic bacteria [15, 16]. During the last two decades, the use of antibiotics in poultry farming has changed, as the utilization of antibiotics as growth promoters is banned or severely restricted in some countries, which caused distinctive differences between countries in regard to prevalence of antibiotic resistance [17, 18].

The objective of our study was to comparatively determine the antibiotic resistance in commensal* E. coli* isolated from chicken's farms between China and Sudan. We utilized WGS and polymerase chain reaction (PCR) techniques to study antimicrobial resistance genes and highlighted the importance of identifying the antibiotics resistance changes in the natural microbiota due to the overuse of antimicrobials by commercial poultry meat producers.

## 2. Materials and Methods

### 2.1. Samples Collection

A total of 500 cloacal swab samples were collected from commercial chicken farms located in Khartoum, Sudan (n=250), and Nanjing, China (n=250), in June, 2016. All samples were swabbed with a sterile cotton swab (Xuzhou Kanger Company, Xuzhou, China). After collection, samples were stored in ice-cold sterile containers during transportation from the farm to the laboratory. The samples collected from Sudan were transferred in LB medium (Sigma Aldrich, China) to the College of Veterinary Medicine Nanjing Agriculture University, China, within two days after collection; for isolation and identification. All the samples collected from China and Sudan were handled at the same time.

### 2.2. Bacterial Isolation

Samples collected from both countries were inoculated (5 *μ*L inoculums) in LB medium and stored at 4°C. Fecal coliform isolates were further characterized by streaking on MacConkey agar (Qingdao hope bio-technology co. ltd., China) and incubated overnight at 37°C. The phylogenetic classification of the bacteria isolates was conducted by multiplex PCR and assigned to the 4 major* E. coli* bacteria groups A, B1, B2, and group D according to the 16S rRNA analysis.

Based on the initial biochemical tests, a total of 250* E. coli* isolates (120 samples from China and 130 samples from Sudan) were selected and stored in sterile containers for further tests.

### 2.3. Antibiotics Agents

All the bacterial isolates were tested for antibiotics resistance including ampicillin, cefotaxime, kanamycin, erythromycin, doxycycline, ciprofloxacin, tetracycline, gentamicin, streptomycin, and spectinomycin. All antimicrobials were purchased from Sigma-Aldrich, China. Antibiotics were prepared and diluted in the LB medium to concentrations ranging from 0.01 *μ*g/mL to 128 *μ*g/mL (Table 1). Isolates which showed resistance to two or more antimicrobial agents were defined as multidrug-resistant.

### 2.4. Antibiotics Testing

Antibiotic susceptibilities of the* E. coli* isolates were determined by minimum inhibitory concentration (MIC) test and were carried out by the agar dilution method according to Clinical and Laboratory Standards Institute (CLSI) recommendations [19].

The MIC was reported as the lowest test concentration extract that showed no visible bacterial growth. The plate wells with a complete inhibition of macroscopic growth were recorded as MIC against the tested strain. The density of the suspension to contain colony forming units (CFU)/mL was adjusted by comparison with a 0.5 McFarland turbidity standard. Tested antibiotics, concentration ranges, and resistance MIC points are listed in Table 1, and* E. coli* ATCC 25922 was used as the control organism.

### 2.5. Detection of Antibiotics Resistance Genes

The PCR amplifications were carried out using PCR primers listed in Table 2, for the detection of 13 different antibiotics resistance genes* ARGs*, such as ampicillin* ampC*, tetracycline* tetA*, kanamycin* pKD13*, ciprofloxacin* acrA*, erythromycin* ermA, ermB*,* ermC*, doxycycline* tetB*, cefotaxime* mphA*, spectinomycin* aadA14*, streptomycin* aadA1*, and gentamicin* aac3-1*,* aac3- III.*

### 2.6. Genomic DNA Extraction

The total bacterial DNA was extracted by using commercial bacterial DNA extraction kit (Omega Co., China) according to the kit manufacturer's instructions. Briefly, 1.5 mL of* E. coli* isolate (grown overnight in LB medium) was pelleted in Eppendorf tube by microcentrifuge at 10,000×g at max speed for 10 min. The supernatant was discarded followed by addition of 100 *μ*L TE buffer and vortexed to completely resuspended the cell pellet. Lysozyme (10 *μ*L) was added and the solution was incubated at 57°C for 10 minutes. Then, 20 *μ*L of proteinase K solution was added, samples were vortexed, and the cell solution was incubated at 55°C shaking water bath for lysis 6 hrs. After the lysis, 5 *μ*L of the RNase was added and the tubes were incubated at room temperature for 5 minutes. This was followed by the addition of 220 *μ*L of BDL buffer; then, samples were vortexed and incubated at 65°C for 10 minutes. The genomic DNA was concentrated by the addition of 220 *μ*L of 100% ethanol, vortexed for 20 seconds, and centrifuged for 1 min at 10,000×g. Then, 500 *μ*L HBC buffer was added and centrifuged for 1 min at 10,000×g for washing. The purified DNA was eluted in a fresh 1.5 mL microcentrifuge tube using 100 *μ*L elution buffer, 171, and kept at room temperature for 5 minutes, and centrifuged for 1 min at 10,000×g. Finally, the DNA concentration was evaluated using nanodrop spectrophotometer before being stored at −20°C.

### 2.7. Genome Annotations and Comparison

DNA sequencing runs of* E. coli* isolates were completed for both genomes of D107 (resistant) strain from China and D4 (sensitive) strain from Sudan. Open reading frames annotation and comparative gene clusters analysis was conducted using the rapid annotation subsystem technology (RAST version 4.0). Gene sequence alignment was performed using Genedoc (version 2.6).

## 3. Results and Discussion

### 3.1. Antibiotics Resistance Testing

Antimicrobial resistance test of all* E. coli* isolates were determined by minimum inhibitory concentration (MIC). Classification and percentage distribution of antibiotic resistance in commensal* E. coli* isolates against 10 different antibiotics is presented in Table 3. All* E. coli* isolates were resistant to at least one antibiotic; these results are consistent with other previous studies on commensal* E. coli*, where commensal* E. coli* exhibited high prevalence of resistance to commonly used antibiotics in livestock [20, 21] (Miles* et al*., 2016). Furthermore, about 75.5% of bacterial isolate were resistant to more than two antibiotics (multidrug-resistant). Among examined individual antibiotics, tetracycline resistance was the highest percentage found in both isolates from China and Sudan at of 80% and 54.4%, respectively. This high rate of resistance in commensal* E. coli* from poultry was also demonstrated in the study of Cizman [22]. Tetracycline is commonly used antibiotic against commensal* E. coli *from chicken farms in the above studied two countries. Another significantly high resistance was found towards ampicillin at 75.2% of isolates from China; however, the isolates from Sudan were significantly lower in percentage (29.2%). This is followed by the resistance to ampicillin, kanamycin, and erythromycin. Moreover, about 62% of the* E. coli* isolates resistant to ampicillin also showed resistance against tetracycline.

The overall phenotypic resistance comparison reveals significant higher rates of antibiotic resistant* E. coli* isolates from China compared to the Sudan isolates except in the ciprofloxacin where the Sudanese isolate showed higher resistance, which might be due to the variation of antibiotics overuse between both countries.

### 3.2. Distribution of* E. coli* Isolates Resistance Genes

**Figure 1:**
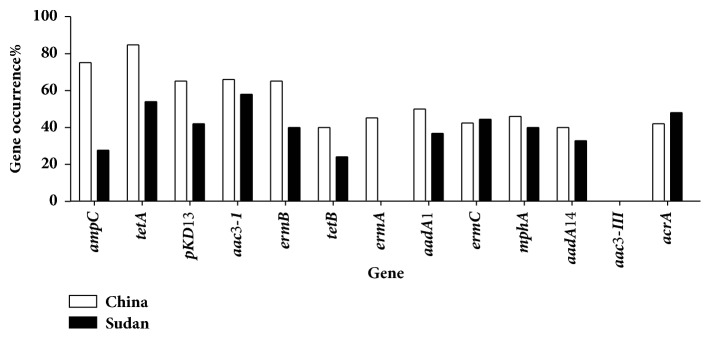
Distribution of resistance genes among commensal* Escherichia coli* isolates, from poultry farms (China and Sudan).

PCR amplifications were performed to detect 13 virulence genes using their appropriate primers. The distribution of identified virulence genes is presented in Figure 1. Tetracycline resistant gene* tetA* was found to be highly distributed in isolates from China and Sudan at 84% and 54%, respectively, which is comparable to some studies reporting higher frequencies of* tetA* and* tetB *[23, 24]. This percentage is followed by the ampicillin resistance gene (*ampC*) at 75% and 27.6%, respectively. The detection of resistance genes was found to be in agreement with the phenotypic resistance data in Table 3, suggesting that these genes play significant role in the tolerance against the tested antibiotic. Moreover, about 72% of the* tetA* resistance isolates were also resistant to* ampC*, and around 35% of isolates possessing* ampC/pKD13* genes in their genome were found to possess* tetA* gene, indicating significantly high prevalence of these multidrug resistance isolates. The occurrence of remaining genes in the genome of tested isolates was present in a range from 34% to 66%, except for erythromycin* ermA* in Sudan isolates and gentamicin (*aac3- III*) in both countries which were not detected, while the* pKD13* genes were identified in 65% and 42% of isolates from China and Sudan, respectively. Therefore, this indicates that local and the geographical localization factors can play a major role in the appearance of some antimicrobial resistance genes. It has been mentioned that sensitive strains harbor antibiotic(s) resistant gene(s) might express this resistant and generate strains that are likely to be resistant to those antibiotics [25].

### 3.3. Comparative Genomes Sequence

**Figure 2:**
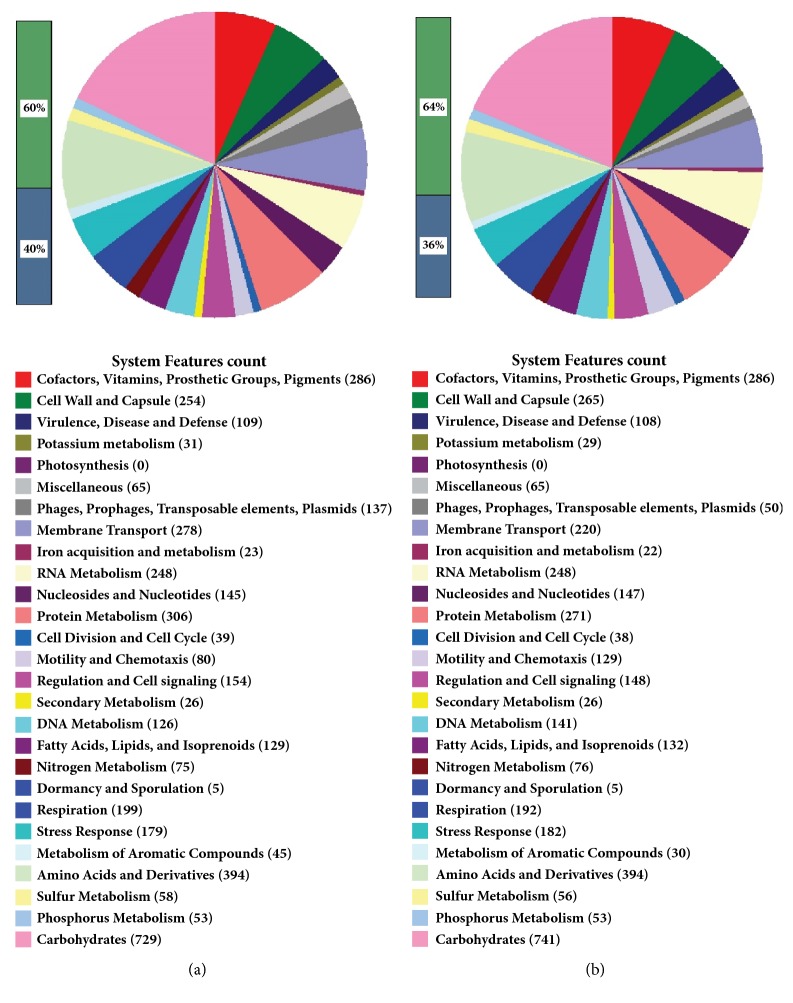
Genomic maps showing deferent group of genes functions categories, annotated by RAST. (a)* E. coli* D107 (China strain); (b)* E. coli* D4 (Sudan strain). The number of shared genes and the number of unique genes and genes shared between two strains are shown.

In order to determine the extent of DNA sequencing coverage, TBLASTN searches were performed against the whole genome sequences of D107 strain from China and D4 strain from Sudan. In general, among these two* E. coli* genomes, the chromosomes of Sudanese strain (more antibiotic sensitive) were found to have smaller genome size (4,742,490 bp) and consisted of 4179 coding DNA sequences (CDS), whereas the Chinese strain has 5,111,357 bp and 5193 CDS.  Figure 2 represents different gene groups categorized by RAST (RAST.nmpdr.org) annotation tool. The genome sequence of commensal* E. coli* strain D107 isolated from China revealed a wide array of antimicrobial resistance genes compared with the whole genome of the* E. coli* D4 strain.

**Figure 3:**
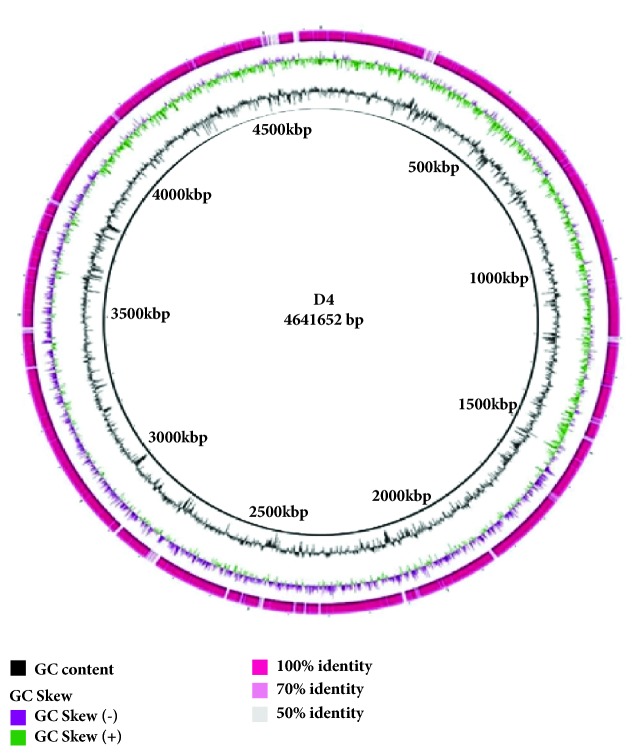
Circular map of D107 and D4 Genomic comparison. The outer circle shows identity position of both genomes. The second circle shows the D107 genome and inner circle shows D4 genome. Gaps seen in D4 indicate that sequence is missing in this isolate but present in D107.

The antimicrobial resistance genes identified in whole genomes sequence of* E. coli* D107 and D4 are listed in Table 4. The comparative sequence analysis of both D107 and D4 genomes (Figure 3) revealed that erythromycin (*ermA*) and cefotaxime gene (*mphD*) antibiotics resistant genes were found in the D107 while being absent in the* E. coli* from the D4 isolate. The missing resistance genes from antimicrobial susceptible strain (D4) suggests that it has various plasmid complement that any antimicrobial resistance determinants were either missing or never obtained. However, quinolone (*qnrA1*) and gentamicin (*aac3- III*) resistance genes were not found in both genomes.

The detection of the differences between the two genomes holds a great potential for understanding the development of risky* E. coli* resistant strains from the Chinese isolate. The chromosomal backbones of the two* E. coli* strains are different as in Figure 3. Although D107 and D4 (both are commensal bacteria) have highly various antimicrobial profiles of high and low susceptibility, respectively, this suggests that transferring exogenous or horizontal genes is a key mechanism for the acquisition of antimicrobial resistance. This is implication is reasonable, since many antimicrobial resistance genes determinants are transmitted by moving plasmids or mobile elements, particularly for gut microorganisms [26].

**Figure 4:**
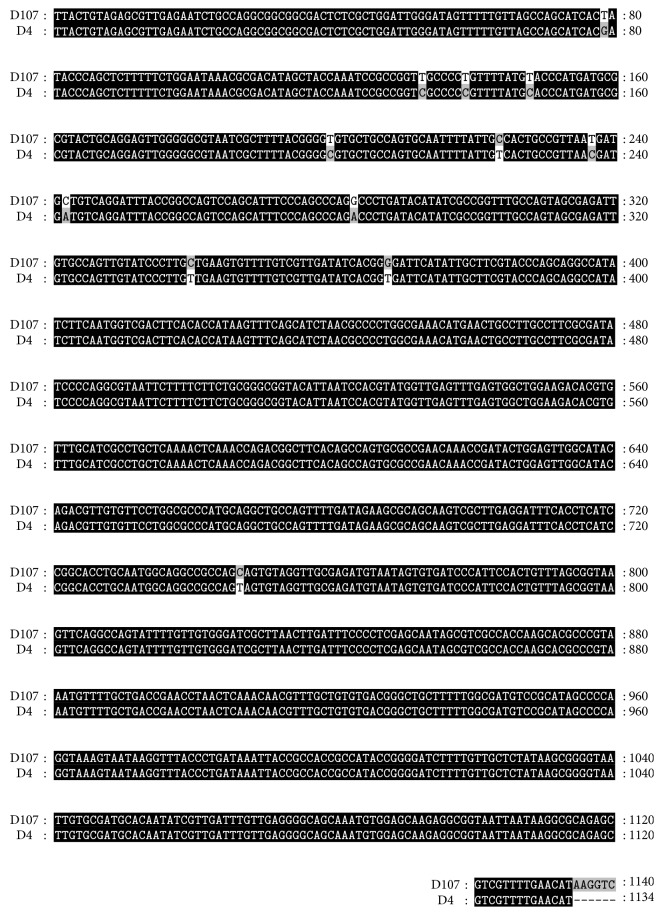
Alignment result of* AmpC *gene in the genomes of China strain (D107) and Sudan strain (D4).

The specific gene sequence comparison in between D107 and D4 strains is exemplified for the* ampC *gene (Figure 4). Although the gene was present in the genome of both strains, the phenotypic resistance varied significantly which could indicate a strong engagement between antibiotics resistance genes and an elevated mutation rate. Moreover, many studies have observed that the acquisition of a mutation provides antibiotic resistance genes [26].

## 4. Conclusion

Herein we report a comparative study on the antibiotic resistance isolates of commensal* E. coli*, evaluating genes associated with antibiotic resistance from Chinese and Sudanese poultry farms. Clear variation in phenotypic resistance patterns was found, as isolates from China were significantly more tolerant to a wide array of antibiotics, which is supported by the detection of specific genes and genome-wide comparative analysis. The results indicate the variations of irrational utilization of these antimicrobial agents in animal farms for treatments or as growing promoters. We are currently exploring the interplay between the genetic makeup of resistant strains and their expressed phenotypic resistance.

## Figures and Tables

**Table 1:** Class, concentration range, and resistant breakpoints of tested antibiotics.

**Class or antibiotics**	**Dilution range tested** **(*μ*g/mL)**	**Resistance breakpoint** **(*μ*g/mL)**
Ampicillin	0.25-32	≥ 32
Tetracycline	1.0-32	≥ 16
Kanamycin	0.25-64	≥ 64
Ciprofloxacin	0.01-15	≥ 04
Erythromycin	0.25-8.0	≥ 08
Doxycycline	1.0-16	≥ 16
Cefotaxime	0.01-5.0	≥ 04
Spectinomycin	1.0-128	≥ 128
Gentamicin	0.25- 8.0	≥ 08
Streptomycin	1.0-128	≥ 128

**Table 2:** Primer sequences and annealing temperatures used in the PCR reactions carried out the virulence genes of *E. coli* isolates in this study.

**Antibiotics **	**Resistances genes**	**Sequence**	**Size (bp)**	**Annealing temp (**°**C)**	**Reference genes**
Ampicillin	*ampC*	(F) AATGGGTTTTCTACGGTCTG (R) GGGCAGCAAATGTGGAGCAA	191		Woodford et al. [10]

Tetracycline	*tet(A)*	(F) GGTTCACTCGAACGACGTCA (R) CTGTCCGACAAGTTGCATGA	577	57	Woodford et al. [10]

Kanamycin	*pKD13*	(F) AGGCTTTTGCTTGAATGTTCCGTCAAGGGATCAC GGGTAGGAGCCACCTTGTGTAGGCTGGAGCTGCTTC (R) GGTCGACGGATCCCCGGAATGTAAGCATCTGTC AGAAAGGCCAGTCTCAAGCGAGGCTGGCCTTTTCTGT			Woodford et al. [10] Wright [9]

Erythromycin	*erm(B)*	(F) GAAAAAGTACTCAACCAAATA (R) AATTTAAGTACCGTTAC	642	45	Woodford et al. [10]
	*erm(A)*	(F) TCTAAAAAGCATGTAAAAGAAA (R) CGATACTTTTTGTAGTCCTTC	533	52	Woodford et al. [10]
	*erm(C)*	(F)TCAAAACATAATATAGATAAA (R)GCTAATATTGTTTAAATCGTCAAT	642	45	Wright [9]

Doxycycline	*tet(B)*	(F) CCTCAGCTTCTCAACGCGTG (R) GCACCTTGCTGATGACTCTT	634	56	Woodford et al. [10]

Spectinomycin	*aadA14*	(F) GTGAGGAGGAGCTTCGCGAG (R) TGCCGCAGGACTCGGAGGTC	642	60	Woodford et al. [10]

cefotaxime	*mph(A) *	(F) GTGAGGAGGAGCTTCGCGAG (R) TGCCGCAGGACTCGGAGGTC	403	60	Wright [9]
	*mph(B)*	(F)GATATTAAACAAGTAATCAGAATAG (R) GCTCTTACTGCATCCATACG	494	58	Wright [9]

Ciprofloxacin	*acrA*	(R) TGCAGAGGTTCAGTTTTGACTGTT (F) CTCTCAGGCAGCTTAGCCCTAA	107		Velicer et al. [11]

Gentamicin	*aac(3)-I *	(F) ACCTACTCCCAACATCAGCC (R) ATATAGATCTCACTACGCGC	169	60	Velicer et al. [11]
	*aac(3)-III*	(F) CACAAGAACGTGGTCCGCTA (R) AACAGGTAAGCATCCGCATC	185	60	Velicer et al. [11]

Streptomycin	*aadA1*	(F) TATCCAGCTAAGCGCGAACT (R) ATTTGCCGACTACCTTGGTC	286	55	Woodford et al. [10]

**Table 3:** Phenotypic pattern of commensal *E. coli* isolates from poultry farms in China and Sudan to 10 antibiotics agents, included in this study.

**Antibiotics**	**Sensitive **%		**Intermediate **%		**Resistant **%	
	**China**	**Sudan**	**China**	**Sudan**	**China**	**Sudan**
Ampicillin	10.0	33.3	15.0	37.5	75.2	29.2
Spectinomycin	20.0	40.2	30.0	22.3	50.0	37.6
Kanamycin	15.0	42.0	20.0	16.0	65.2	42.0
Erythromycin	15.0	37.2	19.8	29.2	65.2	33.6
Cefotaxime	15.0	33.0	39.0	33.3	46.0	33.6
Doxycycline	29.8	45.8	25.0	29.0	45.2	25.2
Tetracycline	5.0	29.1	15.0	16.5	80.0	54.4
Ciprofloxacin	20.0	25.0	24.8	25.0	46.0	50.0
Gentamicin	24.4	30.0	34.0	37.0	41.6	33.6

**Table 4:** Inventory of antibiotic resistance genes identified in whole *E. coli* genomes (D107 and D4).

**Antibiotics genes**	**D 107**	**D 4**
*ampC*	1	1
*tetA*	1	1
*pKD13*	1	1
*aac3-1*	1	1
*ermA*	1	0
*qnrA1*	0	0
*ermB*	1	1
*tetB*	1	1
*aadA1*	1	1
*gyr*	1	1
*mphA*	1	1
*aadA14*	1	1
*aac3- III*	0	0
*acrA*	1	1
*mphD*	1	0

## Data Availability

The data used to support the findings of this study are available from the corresponding author upon request.
